# Severe eosinophilia and risk of major disease and mortality: A nationwide cohort study of children and adults

**DOI:** 10.1016/j.jacig.2026.100654

**Published:** 2026-02-04

**Authors:** Shay Nemet, Daniel Elbirt, Ramon Cohen, Keren Mahlab-Guri, Vered Shkalim Zemer, Ilan Asher, Aviv Talmon, Limor Rubin, Yaarit Ribak, Eyal Ben-Dori, Inon Sarig, Nur Sagi, Ruslan Sergienko, Yuval Tal, Oded Shamriz

**Affiliations:** a Allergy and Clinical Immunology Unit, Kaplan Medical Center, Faculty of Medicine, Hebrew University of Jerusalem, Jerusalem, Israel; b Faculty of Medical and Health Sciences, Tel Aviv University, Tel Aviv, Israel; c Clalit Health Services, Dan-Petach Tikva District, Petach Tikva, Israel; d Allergy and Clinical Immunology Unit, Department of Medicine, Hadassah Medical Organization, Faculty of Medicine, Hebrew University of Jerusalem, Jerusalem, Israel; e Department of Health Policy and Management, Faculty of Health Sciences, Ben-Gurion University of the Negev, Beer-Sheva, Israel; f The Lautenberg Center for Immunology and Cancer Research, Institute of Medical Research Israel-Canada (IMRIC), Faculty of Medicine, Hebrew University of Jerusalem, Jerusalem, Israel

**Keywords:** Severe eosinophilia, population-based, nationwide, long-term, epidemiology

## Abstract

**Background:**

Severe eosinophilia (SE), defined as greater than 5000 cells/μL, is uncommon but may indicate serious underlying disease. Its long-term prognostic implications across age groups remain unclear.

**Objectives:**

We sought to investigate the long-term risk of morbidity and all-cause mortality in children and adults with SE.

**Methods:**

We conducted a nationwide, population-based, matched cohort study using data from Clalit Health Services, Israel (2000-2023). Individuals with SE (n = 3822) were matched 1:10 to subjects without SE with normal eosinophil counts (<500/μL; n = 39,005). Five-year risks of cancer, autoimmune disease, thromboembolic events, allergic disorders, and all-cause mortality were assessed using multivariable Cox proportional hazards models.

**Results:**

The study included 42,827 participants, both with and without SE. Among the 29,289 adults, 2,497 had SE and 26,792 did not. Among the 13,538 children, 1,325 had SE and 12,213 did not. Among adults, SE was associated with increased risk of hematologic malignancy (hazard ratio [HR], 2.86; 95% CI, 1.88-4.36), autoimmune disease (HR, 1.64; 95% CI, 1.38-1.95), thromboembolic events (HR, 1.91; 95% CI, 1.45-2.53), and mortality (HR, 2.20; 95% CI, 1.84-2.62). In children, SE predicted solid tumors (HR, 14.24; 95% CI, 2.39-85.00), autoimmune disease (HR, 2.48; 95% CI, 1.97-3.12), allergic disorders (HR, 1.45; 95% CI, 1.26-1.67), and markedly increased mortality (HR, 7.95; 95% CI, 3.23-19.56).

**Conclusions:**

Severe eosinophilia is a strong prognostic marker in both children and adults, associated with malignancy, autoimmune and thromboembolic disease, and premature death. Recognition of SE should prompt comprehensive evaluation and close follow-up in general and specialty caregivers.

Eosinophils play a multifaceted role in maintaining immune balance and host defense. They contribute to protection against parasitic infections and participate in various physiological processes, including hemostasis, regulation of metabolic functions, immunomodulation, and control of carcinogenesis.^1^^,^^2^

Eosinophilia is defined as an absolute eosinophil count greater than 500 cells/μL and is classified as mild (500-1500 cells/μL), moderate (1500-5000 cells/μL), or severe (SE; >5000 cells/μL). This hematologic classification should be distinguished from *hypereosinophilia*, defined as an absolute eosinophil count exceeding 1500 cells/μL on at least 2 occasions separated by a minimum of 2 weeks, and from *tissue eosinophilia*, which denotes organ-specific eosinophilic infiltration and inflammation, with diagnostic thresholds that vary by tissue type. *Hypereosinophilic syndrome* represents a pathologic condition in which persistent hypereosinophilia leads to end-organ damage attributable to eosinophil activity, after exclusion of other underlying causes.^3^

Causes of SE vary geographically. In tropical and developing regions, parasitic infections, such as strongyloidiasis, predominate,^4^ whereas in industrialized countries with lower parasitic burden, allergic disorders and drug reactions are more common.^4^^,^^5^

SE can be suggestive of specific conditions. For example, SE is uncommon in patients with asthma, but when present, it may indicate underlying conditions, such as eosinophilic granulomatosis with polyangiitis.^2^^,^^4^ In addition, there are other rare etiologies for SE, some with potentially life-threatening outcomes, including drug reaction with eosinophilia and systemic symptoms syndrome^6^ and immune checkpoint inhibitors–induced eosinophilic disorders.^7^

Eosinophils exert tissue damage and drive chronic inflammation primarily through the release of cytotoxic proteins such as eosinophil peroxidase, eosinophil cationic protein, and major basic protein.^2^ Beyond their inflammatory effects, eosinophils also play a dual role in tumor regulation, either promoting or inhibiting tumor growth. This tumor-modulating activity is shaped by the tumor microenvironment (TME) and the balance among T_H_1, T_H_2, and T_H_17 responses.^8^

The clinical implications of SE have been described in smaller cohorts. A retrospective study from Boston and Vermont reported that 39% of 193 patients with SE had underlying malignancies, whereas only 3% had helminthic infections.^9^ In a single-center study from China, clinical workup was noted in only 63 of 621 (10.1%) patients with moderate-to-severe eosinophilia.^10^ Similarly, in another study published by our group, SE was overlooked or not investigated in 28% of 239 patients, even though 42% of patients with SE had underlying malignancies.^11^ Collectively, these findings highlight that, despite previous single-center analyses, the broader epidemiologic and prognostic significance of SE, particularly across different age groups, remains insufficiently characterized.

In this population-based study, we investigate the long-term risk of malignancies, thromboembolic disorders (TEDs), and autoimmune disorders (AIDs) in children and adults with SE.

## Methods

### Study population, design, and eligibility criteria

This study used a retrospective cohort design within a target trial emulation framework, structuring the analysis as if it were a hypothetical randomized trial by explicitly defining eligibility criteria, exposure, start of follow-up, outcomes, and the analysis plan. This approach was used to evaluate SE as a risk factor for the development of various clinical conditions over a 5-year postindex period.^12^^,^^13^ For the purpose of this study, SE was defined according to hematologic criteria as an absolute eosinophil count exceeding 5000 cells/μL, consistent with definitions used in previous studies and reports.^11^^,^^14^^,^^15^

Cases (n ≈ 3000) were identified from the electronic medical records database of Clalit Health Services (CHS), the largest health maintenance organization in Israel, providing coverage to more than 4.5 million individuals. The CHS network includes approximately 1500 clinics and 14 hospitals nationwide. No-SE controls (<500 cells/μL) were selected from the same database at a 1:10 matching ratio, matched by birth year, sex, and ethnicity, and restricted to individuals whose maximum eosinophil count never exceeded 500 cells/μL. The index date was defined as the date of SE diagnosis for each case, with matched non-SE subjects assigned the same index date.

Children and adults entered the analytical follow-up period after a 6-month lag from the index date. This lag period was incorporated to mitigate reverse causation and exclude conditions likely preexisting but undiagnosed at the time of SE detection. Patients were followed for up to 5 years, with follow-up ending at the earliest occurrence of a study outcome, death, loss to follow-up, or completion of the 5-year period.

Patients were excluded if they lacked critical covariate data, including body mass index (BMI) or Charlson comorbidity index (CCI) scores, or if they had been diagnosed with any of the study outcomes before or within the 6-month lag period (outcome-specific washout periods). Separate analytic cohorts were constructed for each clinical outcome, ensuring the exclusion of prevalent cases to enable accurate incidence estimation.

The primary outcomes included the development of cancer, AID, TED, allergies, immunodeficiency, autoinflammatory disorders, and all-cause mortality during the 5-year postindex follow-up period.

Baseline covariates included age, sex, ethnicity, socioeconomic score (SES), BMI, CCI, and preexisting clinical comorbidities. Data were extracted from the CHS using the Clalit Research Data Sharing Platform, powered by MDClone (https://www.mdclone.com).

### Statistical analysis

Analyses were structured and aligned with the target trial emulation methodology. Initial data preparation involved detailed verification of matching accuracy, consistency of demographic and clinical covariates, and rigorous validation of clinical outcome ascertainment. Data sets were explicitly structured to ensure that patients were free of the respective outcomes during the predefined washout and lag periods, thereby enabling accurate incidence estimation.

Baseline characteristics of the study population were summarized using standard statistical methods. Continuous variables were presented as means ± SD or medians (interquartile ranges), as appropriate, and categorical variables as frequencies and percentages. Differences between groups at baseline were assessed using standardized mean differences (SMDs), with an SMD exceeding 0.1 considered indicative of meaningful imbalance.

The primary analysis used time-to-event methods beginning at the end of the 6-month lag period. Kaplan-Meier survival curves were constructed for each outcome, and log-rank tests were used to evaluate differences in cumulative incidence between the eosinophilia and non-SE groups. Multivariable Cox proportional hazards models were used to estimate hazard ratios (HRs) and 95% CI, adjusting for potential confounders including age, sex, nationality, BMI, and CCI scores. The proportional hazards assumption was assessed through graphical inspection and Schoenfeld residual tests, with no significant violations detected.

Confounding was addressed using propensity score methods. Propensity scores estimating the probability of SE were derived from logistic regression models incorporating baseline covariates. Matching was performed using nearest-neighbor matching, with a caliper width of 0.2 SDs of the logit of the propensity score. Balance was assessed using SMDs, with values below 0.1 indicating adequate covariate balance.

Sensitivity analyses included evaluation of alternative lag periods (3 and 12 months) to assess the robustness of associations under varying assumptions about disease latency and detection bias. Additional analyses excluded outcomes diagnosed within the first-year postindex to further mitigate residual reverse causation.

A prespecified subgroup analysis was conducted to assess heterogeneity in the association between eosinophilia and outcomes based on eosinophil count severity (5,000-10,000 vs >10,000 cells/μL). Interaction terms between eosinophilia status and subgroup variables were tested within Cox proportional hazards models.

All statistical analyses were performed using SPSS for Windows, version 29.0 (IBM Corp, Armonk, NY), and R Statistical Software, version 4.2,3 (R Foundation for Statistical Computing,Vienna, Austria) using the following packages: survival, MatchIt, tableone, and cmprsk.

### Ethical approval of the study

The study protocol was approved by CHS Institutional Review Board (IRB# KMC-0106-23). Confidentiality complied with ethical guidelines for human subject research. Because of the retrospective nature of the study, a waiver from obtaining informed consent was given by the IRB.

## Results

### Demographic and baseline characteristics

Table I summarizes the baseline characteristics of the adult and pediatric cohorts. The adult cohort comprised 29,289 individuals, including 13,734 (46.9%) females and 15,555 (53.1%) males. Severe eosinophilia was present in 2,497 individuals (8.5%), and 26,792 matched non-SE subjects with normal eosinophil counts (<500/μL) were identified. Mean age at the index date and age distribution did not differ between groups. Non-SE controls had higher rates of medium SES and elevated BMI (*P <* .001 for both), whereas patients with SE had a higher CCI (*P <* .001), reflecting greater comorbidity burden. Most subjects were Jewish (81.92% of non-SE subjects vs 80.18% of patients with SE).

**Table I:** Sociodemographic characteristics of the study’s population

Characteristics			Adults (≥18 y)							Children (<18 y)						
			Total		Non-SE subjects		Severe eosinophilia		*P* value	Total		Non-SE subjects		Severe eosinophilia		*P* value
			N	%	N	%	N	%		N	%	N	%	N	%	
No. of subjects			29,289	100	26,792	91.5	2,497	8.5	—	13,538	100.0	12,213	90.2	1,325	9.8	—
Sex	Female		13,734	46.89	12,575	46.94	1,159	46.42	.619	5,156	38.09	4,647	38.05	509	38.42	.795
	Male		15,555	53.11	14,217	53.06	1,338	53.58		8,382	61.91	7,566	61.95	816	61.58	
SES (3L)	Low		8,201	28.00	7,482	27.93	719	28.79	<.001∗	5,191	38.34	4,677	38.30	514	38.79	.860
	Medium		11,198	38.23	10,352	38.64	846	33.88		4,844	35.78	4,379	35.86	465	35.09	
	High		9,890	33.77	8,958	33.44	932	37.32		3,503	25.88	3,157	25.85	346	26.11	
Ethnicity	Jewish		23,949	81.77	21,947	81.92	2,002	80.18	.072	8,920	65.89	8,053	65.94	867	65.43	.935
	Arab		4,090	13.96	3,704	13.83	386	15.46		3,681	27.19	3,316	27.15	365	27.55	
	Other		1,250	4.27	1,141	4.26	109	4.37		937	6.92	844	6.91	93	7.02	
Born in Israel	No		13,329	45.51	12,174	45.44	1,155	46.26	.433	558	4.12	483	3.95	75	5.66	.003∗
	Yes		15,960	54.49	14,618	54.56	1,342	53.74		12,980	95.88	11,730	96.05	1,250	94.34	
Age at index date (categorized, y)	Adults	Children	4,524	15.45	4,113	15.35	411	16.46	.337	8,305	61.35	7,451	61.01	854	64.45	.049∗
	18-30	0-3														
	31-64	3-12	13,445	45.90	12,309	45.94	1,136	45.49		3,571	26.38	3,252	26.63	319	24.08	
	≥65	12-18	11,320	38.65	10,370	38.71	950	38.05		1,662	12.28	1,510	12.36	152	11.47	
Age at index date (y), mean ± SD			56 ± 20		56 ± 20		56 ± 20		.472	4.22 ± 5.00		4.26 ± 5.01		3.87 ± 4.95		.007∗
BMI (kg/m^2^), mean ± SD			27.0 ± 5.2		27.1 ± 5.3		26.3 ± 5.0		<.001∗	17.1 ± 3.3		17.1 ± 3.3		16.7 ± 3.3		<.001∗
CCI, mean ± SD			2.6 ± 2.7		2.5 ± 2.6		3.8 ± 3.4		<.001∗	0.1 ± 0.4		0.1 ± 0.3		0.3 ± 0.9		<.001∗

∗ The χ^2^ statistic is significant at the .05 level.

The pediatric cohort included 13,538 children, of whom 1,325 had SE and 12,213 were matched non-SE subjects. Males predominated in both groups (61.58% in SE, 61.95% in controls; no significant difference). Children with SE were younger than their non-SE counterparts (mean age, 3.87 ± 4.95 vs 4.26 ± 5.01 years; *P =* .007), with most being younger than 3 years. As in adults, BMI was higher among non-SE subjects (*P <* .001), whereas CCI was higher in the SE group (*P <* .001). SES did not differ significantly between groups.

### Prevalence of preexisting conditions in pediatric and adult populations

We first evaluated the prevalence of preindex comorbidities in each cohort (see Table E1 in this article’s Online Repository at www.jaci-global.org). In both adults and children, individuals with SE had significantly higher rates of cancer (any, solid, and hematologic malignancies), AIDs, inborn errors of immunity, TEDs, allergies, adrenal insufficiency, and prior splenectomy *(P <* .001 for each condition in both cohorts; adults: *P =* .040 for adrenal insufficiency). Rates of hematologic malignancies were markedly higher in adults (19.62% vs 1.60%, respectively) and children (4.62% vs 0.007%) with SE compared with non-SE subjects.

Autoinflammatory disorders were more frequent in adults with SE compared with non-SE subjects (*P =* .018) but showed no significant difference in children (*P =* .559). The distribution of preindex solid and hematologic malignancies in the adult and pediatric cohorts is presented in Tables E1 to E4 (in the Online Repository available at www.jaci-global.org). Among hematologic malignancies, leukemia was the most prevalent preindex diagnosis in both adults and children (Tables E2 and E4). For solid tumors, skin cancers were most common in adults, whereas other solid tumor types predominated in children (Tables E3 and E5).

### Adults with SE are at higher risk of hematologic malignancies, AIDs, and TEDs

**Fig 1:**
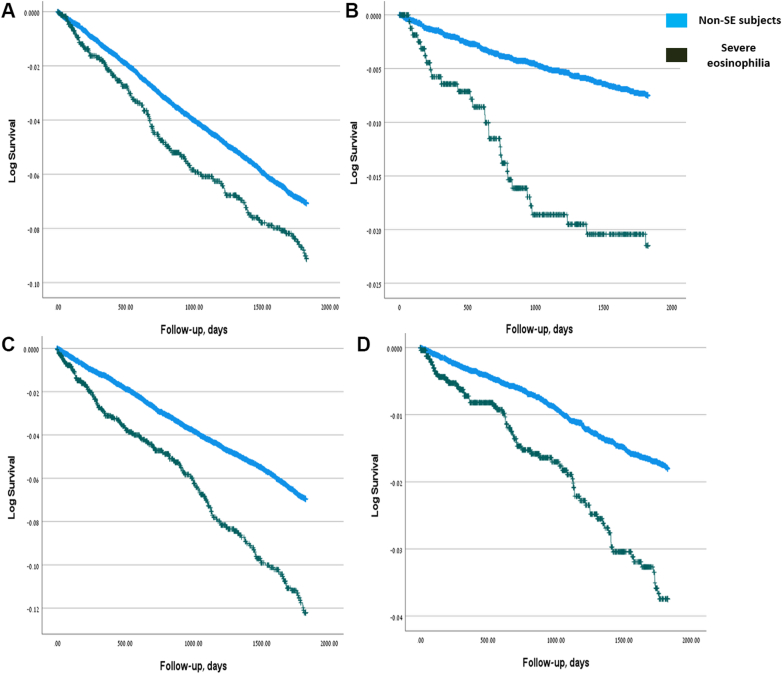
Cumulative incidence of cancer, AIDs, and thromboembolic events in adults with SE. Kaplan-Meier curves indicating the cumulative incidence of the outcome over time among adults with SE, based on univariable analysis. **A,** Cancer (any), **B,** hematologic cancer, **C,** autoimmune diseases, and **D,** TEDs.

Next, we used a multivariable Cox regression model to evaluate the risk of developing various outcomes. Results for the adult cohort are presented in Table II. Adults with SE had a significantly higher risk of developing cancer during follow-up (HR, 1.387; 95% CI, 1.146-1.679; *P <* .001). When stratified by cancer type, the increased risk was significant only for hematologic malignancies (HR, 2.863; 95% CI, 1.881-4.359; *P <* .001). Hematologic malignancies in the SE group consisted mainly of leukemia (n = 14; 48.3%), with additional cases of lymphoma and multiple myeloma (Table E2). Adults with SE also had a higher risk of developing AID (HR, 1.640; 95% CI, 1.379-1.950; *P <* .001) and TED (HR, 1.913; 95% CI, 1.446-2.530; *P <* .001). In contrast, SE was not associated with an increased risk of allergies in adults. A repeated multivariable analysis with adjustment for elevated C-reactive protein levels (>0.5 mg/dL) did not increase HR to develop hematologic malignancies in adults (Table E6), thus indicating that SE by itself, rather than a coexisting inflammatory state, was associated with increased risk of hematologic malignancies. Kaplan-Meier curves illustrating the univariable risk of developing cancer, hematologic malignancies, AID, and TED are presented in Fig 1.

**Table II:** Multivariable Cox regression model assessing the risk of developing various outcomes during follow-up in adults with SE

Outcome/variable	Cancer (any)		Solid cancer		Hematologic cancer		AID		Allergy (any)		TEDs	
	HR (95% CI)	*P* value	HR (95% CI)	*P* value	HR (95% CI)	*P* value	HR (95% CI)	*P* value	HR (95% CI)	*P* value	HR (95% CI)	P value
Study group	1.387 (1.146-1.679)	.001∗	1.201 (0.967-1.492)	.097	2.863 (1.881-4.359)	<.001∗	1.640 (1.379-1.950)	<.001∗	1.070 (0.930-1.230)	.344	1.913 (1.446-2.530)	<.001∗
Age at index date (y)	1.048 (1.043-1.05)	<.001∗	1.051 (1.045-1.057)	<.001∗	1.036 (1.022-1.051)	<.001∗	1.008 (1.033-1.052)	.001∗	1.003 (1.000-1.006)	.024∗	1.038 (1.028-1.047)	<.001∗
Sex	1.132 (1.020-1.257)	.020∗	1.173 (1.050-1.311)	.005∗	0.880 (0.648-1.195)	.415	0.805 (0.725-0.894)	<.001∗	0.761 (0.711-0.815)	<.001∗	0.753 (0.622-0.912)	.004∗
SES (3L)	—	<.001∗	—	<.001∗	—	.803	—	.002∗	—	.039∗	—	.021∗
SES (3L) (medium)	1.102 (0.952-1.275)	.192	1.117 (0.954-1.308)	.169	1.011 (0.689-1.484)	.956	1.160 (1.001-1.345)	.049∗	1.113 (1.015-0.219)	.023∗	1.155 (0.905-1.474)	.247
SES (3L) (high)	1.476 (1.272-1.712)	<.001∗	1.565 (1.335-1.834)	<.001∗	0.894 (0.578-1.384)	.615	1.322 (1.132-1.544)	<.001∗	1.126 (1.019-1.243)	.019∗	0.837 (0.634-1.105)	.210
Ethnicity	—	.078	—	.002∗		.076		.109	—	.041∗	—	.219
Ethnicity (Arabic)	0.777 (0.624-0.967)	.024∗	0.638 (0.496-0.821)	<.001∗	1.664 (1.015-2.727)	.043∗	0.911 (0.754-1.100)	.332	1.130 (1.007-1.268)	.038∗	1.353 (0.962-1.904)	.082
Ethnicity (other)	0.943 (0.678-1.313)	.729	0.985 (0.697-1.391)	.930	0.625 (0.197-1.980)	.425	0.675 (0.456-0.999)	.049∗	0.879 (0.704-1.098)	.256	1.111 (0.635-1.944)	.713
Born in Israel	1.082 (0.952-1.231)	.227	1.093 (0.954-1.252)	.200	1.011 (0.685-1.493)	.955	1.176 (1.032-1.332)	.011∗	1.228 (1.131-1.334)	<.001∗	0.989 (0.778-1.258)	.930
BMI	0.998 (0.988-1.008)	.703	0.997 (0.986-1.008)	.556	1.006 (0.978-1.034)	.669	1.013 (1.003-1.022)	.012∗	1.010 (1.003-1.016)	.004∗	1.024 (1.008-1.041)	.004∗
CCI	1.058 (1.024-1.092)	.001∗	1.053 (1.018-1.090)	.003∗	1.089 (0.997-1.189)	.058	1.046 (1.011-1.083)	.009∗	1.096 (1.070-1.122)	<.001∗	1.111 (1.058-1.166)	<.001∗
Cancer (preindex)	—		—		—		1.058 (0.897-1.247)	.502	1.093 (0.973-1.228)	.134	1.178 (0.924-1.503)	.186
AID (preindex)	0.984 (0.854-1.133)	.818	0.967 (0.831-1.126)	.669	0.000 (0.000---)	.658	—		1.348 (1.221-1.487)	<.001∗	1.093 (0.864-1.382)	.458
Autoinflammatory disease (preindex)	1.591 (0.712-3.556)	.258	1.882 (0.842-4.210)	.124	2.247 (0.312-16.172)	.936	1.901 (0.947-3.815)	.071	1.403 (0.829-2.373)	.207	1.281 (0.317-5.171)	.728
IEI (preindex)	1.190 (0.493-2.868)	.699	1.064 (0.398-2.845)	.901	0.986 (0.709-1.372)	.421	0.502 (0.125-2.010)	.330	1.504 (0.716-3.161)	.281	2.148 (0.687-6.714)	.188
Any allergy (preindex)	1.002 (0.894-1.123)	.971	1.003 (0.888-1.133)	.960	1.460 (0.706-3.019)	.935	1.285 (1.147-1.440)	<.001∗	—		1.086 (0.888-1.327)	.422
Thromboembolic disease (preindex)	0.848 (0.607-1.184)	.333	0.757 (0.518-1.104)	.148	2.863 (1.881-4.359)	.308	1.014 (0.713-1.442)	.937	1.096 (0.839-1.431)	.501	—	

*IEI*, Inborn errors of immunity.

∗ The χ^2^ statistic is significant at the .05 level.

### Children with SE exhibit elevated risk of solid tumors, AID, and allergies

**Fig 2:**
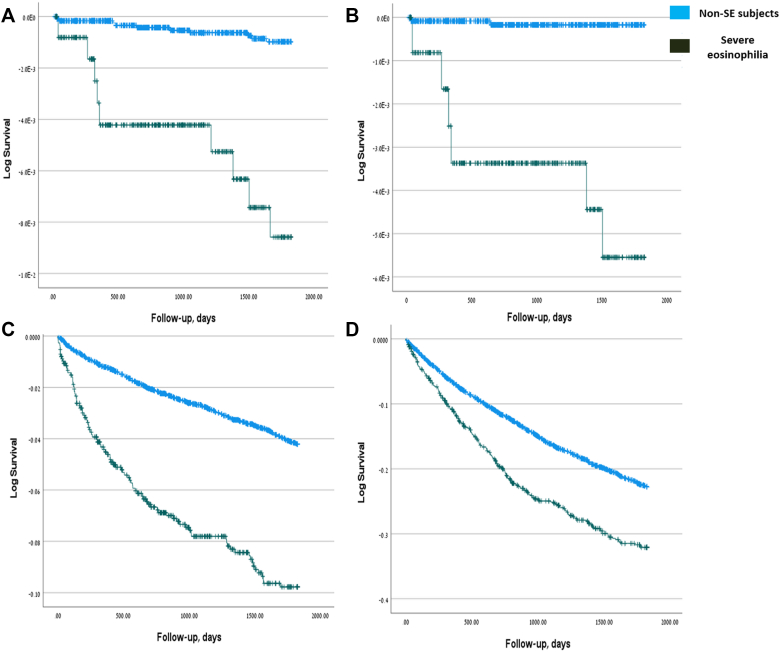
Cumulative incidence of cancer, AIDs, and allergies in children with SE. Kaplan-Meier curves indicating the cumulative incidence of the outcome over time among children with SE, based on univariable analysis. **A,** Cancer (any), **B,** solid cancer, **C,** autoimmune diseases, and **D,** allergies (any).

A similar analysis was performed for the pediatric cohort (Table III). As in adults, children with SE had a significantly higher 5-year risk of developing cancer (HR, 4.954; 95% CI, 1.764-13.917; *P =* .002). In children, however, this increased risk was significant only for solid tumors (HR, 14.241; 95% CI, 2.386-84.995; *P =* .004). Distribution of postindex solid tumors in children included skin malignancies in 2 children (25%) from the SE group and 2 (100%) children from the non-SE control (Table E5). Other Children with SE were also at greater risk of being diagnosed with AID (HR, 2.481; 95% CI, 1.972-3.123; *P <* .001). Notably, and in contrast to adults, SE in children was also associated with an increased risk of allergic disorders (HR, 1.450; 95% CI, 1.257-1.672; *P <* .001). Kaplan-Meier curves representing the risk of developing cancer, solid tumors, AID, and allergic disorders in the pediatric cohort are presented in Fig 2.

**Table III:** Multivariable Cox regression analysis of outcome risks during follow-up in children with SE

Outcome/variable	Cancer (any)		Solid cancer		Hematologic cancer		AID		Allergy (any)	
	HR (95% CI)	*P* value	HR (95% CI)	*P* value	HR (95% CI)	*P* value	HR (95% CI)	*P* value	HR (95% CI)	*P* value
Study group	4.954 (1.764-13.917)	.002∗	14.241 (2.386-84.995)	.004∗	2.171 (0.493-9.553)	.305	2.481 (1.972-3.123)	<.001∗	1.450 (1.257-1.672)	<.001∗
Age at index date (y)	0.925 (0.820-1.044)	.208	0.942 (0.758-1.172)	.594	0.918 (0.789-1.069)	.272	0.961 (0.939-0.984)	.001∗	0.934 (0.922-0.946)	<.001∗
Sex	0.253 (0.093-0.688)	.007∗	0.672 (0.144-3.133)	.613	0.120 (0.026-0.563)	.007∗	0.967 (0.808-1.156)	.710	0.977 (0.893-1.070)	.618
SES (3L)	—	.429	—	.307	—	.950	—	.992	—	.016∗
SES (3L) (medium)	1.806 (0.595-5.483)	.297	3.361 (0.657-17.204)	.146	0.968 (0.213-4.410)	.967	0.997 (0.796-1.250)	.981	1.131 (1.007-1.271)	.038∗
SES (3L) (high)	0.921 (0.190-4.467)	.918	0.011 (0.000-0.000)	.685	0.771 (0.131-4.525)	.773	1.011 (0.784-1.305)	.932	1.207 (1.060-1.375)	.005∗
Ethnicity	—	.677	—	.411	—	.982	—	.102	—	.071
Ethnicity (Arabic)	1.641 (0.546-4.928)	.378	2.882 (0.589-14.094)	.191	0.860 (0.181-4.093)	.850	0.813 (0.639-1.035)	.093	0.869 (0.767-0.984)	.027∗
Ethnicity (other)	0.000 (0.000---)	.978	0.009 (0.000----)	.815	0.000	.984	0.690 (0.443-1.073)	.100	0.883 (0.713-1.093)	.253
Born in Israel	—		—		—		3.042 (1.505-6.149)	.002∗	1.572 (1.220-2.026)	<.001∗
BMI	1.116 (0.993-1.254)	.064	1.096 (0.860-1.396)	.458	1.122 (0.979-1.286)	.098	1.018 (0.986-1.051)	.275	1.041 (1.024-1.058)	<.001∗
CCI	1.647 (0.839-3.235)	.147	1.426 (0.339-6.000)	.628	2.133 (0.968-4.702)	.060	0.998 (0.782-1.273)	.987	1.218 (1.036-1.431)	.017∗
Cancer (preindex)	—		—		—		0.700 (0.213-2.296)	.556	0.416 (0.178-0.970)	∗.042
AID (preindex)	1.332 (0.374-4.745)	.658	0.514 (0.051-5.207)	.574	3.826 (0.905-16.181)	.068	—		0.940 (0.762-1.160)	.566
Autoinflammatory disease (preindex)	0.000 (0.000---)	.992	0.004 (0.000---)	.946	0.000 (0.000---)	.998	1.003 (0.140-7.170)	.998	2.278 (0.945-5.493)	.067
IEI (preindex)	13.217 (3.380-51.682)	.000∗	26.034 (4.655-145.582)	.000	0.000 (0.000---)	.996	1.242 (0.459-3.361)	.669	1.979 (1.098-3.569)	.023∗
Any allergy (preindex)	2.694 (0.971-7.476)	.057	1.822 (0.343-9.679)	.481	3.152 (0.841-11.821)	.089	1.016 (0.802-1.288)	.892	—	
Thromboembolic disease (preindex)	6.089 (0.639-58.020)	.116	12.469 (0.965-161.034)	.053	0.000 (0.000---)	.998	5.258 (0.685-40.379)	.111	1.881 (0.441-8.022)	.393

*IEI*, Inborn errors of immunity.

∗ The χ^2^ statistic is significant at the .05 level.

### Risk of adverse outcomes is mostly unaffected by the site of sampling

Blood sampling on the index date was performed in both hospitalized patients and individuals attending one of the CHS primary care outpatient clinics. The site of sampling, like the CCI, may reflect both the severity of the patient’s clinical condition and the presence of underlying comorbidities, which could potentially influence our results. To address this, we conducted a separate analysis comparing subjects whose blood was drawn during hospitalization with those whose samples were obtained in the outpatient setting (Table E7). In both children and adults, and for all outcomes assessed, most subjects had their samples collected during hospitalization rather than in the ambulatory setting. Interestingly, in adults there were no significant differences in the frequencies of postindex outcomes between hospitalized and outpatient groups, suggesting that the site of eosinophil sampling did not influence results. Similar findings were observed in children, except for AID and cancer, where hospitalized children showed significantly higher frequencies compared with outpatients (*P* = .044 and *P* = .036, respectively).

### SE is associated with increased all-cause mortality in children and adults

**Fig 3:**
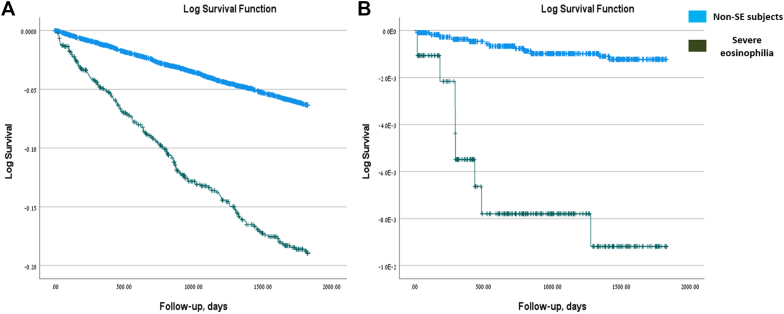
Cumulative incidence of all-cause mortality in children and adults with SE. Kaplan-Meier curves indicating the cumulative incidence of all-cause mortality over time among children and adults with SE, based on univariable analysis. **A,** Adults. **B,** Children.

Finally, we evaluated all-cause mortality in children and adults with SE. Previous studies have suggested that SE may increase mortality risk.^16^ To assess this, we performed a multivariable Cox regression analysis of all-cause mortality during follow-up in both pediatric and adult cohorts (Table IV). Adjustments were made for demographic variables, including age at index date, sex, SES, ethnicity, country of birth (Israel vs other), BMI, and CCI. Notably, both children and adults with SE demonstrated significantly increased all-cause mortality. In adults, SE was associated with an HR of 2.199 (95% CI, 1.844-2.621), whereas in children the risk was markedly higher (HR, 7.949; 95% CI, 3.230-19.564; *P <* .001 for both cohorts). The risk of all-cause mortality during follow-up in adults and children with SE is displayed by Kaplan-Meier curve in Fig 3. These findings indicate that, independent of baseline demographic characteristics, the presence of SE substantially increases the risk of death from all causes in both children and adults.

**Table IV:** Multivariable Cox regression analysis of all-cause mortality risk during follow-up in adults and children with SE

Outcome/variable	All-cause mortality: Adults		All-cause mortality: Children	
	HR (95% CI)	*P* value	HR (95% CI)	*P* value
Study group	2.199 (1.844-2.621)	<.001∗	7.949 (3.230-19.564)	<.001∗
Age at index date (y)	1.081 (1.074-1.089)	<.001∗	0.954 (0.848-1.073)	.431
Sex	1.336 (1.181-1.510)	<.001∗	2.566 (0.857-7.683)	.092
SES (3L)	—	.001∗	—	.669
SES (3L) (medium)	0.824 (0.713-0.952)	.009∗	1.613 (0.562-4.634)	.374
SES (3L) (high)	0.730 (0.620-0.859)	<.001∗	1.524 (0.354-6.570)	.572
Ethnicity	—	.027∗	—	.084
Ethnicity (Arabic)	1.389 (1.093-1.765)	.007∗	3.534 (1.163-10.738)	.026∗
Ethnicity (other)	1.062 (0.770-1.464)	.715	0.000 (0.000---)	.972
Born in Israel	0.865 (0.723-1.034)	.110	0.353 (0.073-1.709)	.196
BMI	0.981 (0.969-0.994)	.003∗	1.058 (0.915-1.222)	.447
CCI	1.284 (1.249-1.319)	<.001∗	0.444 (0.065-3.056)	.410

*IEI*, Inborn errors of immunity.

∗ The χ^2^ statistic is significant at the .05 level.

## Discussion

In this nationwide cohort, SE was linked with markedly increased 5-year risks of malignancy, AID, TED, and all-cause mortality. Cancer patterns differed by age: hematologic malignancies predominated in adults, whereas solid tumors were more common in children. Excess mortality was striking—8-fold in children, 2-fold in adults.

Higher CCI scores were seen in children and adults with SE. To avoid confounding, we took a lag period and adjusted to CCI. However, increased CCI in subjects with SE may not be a confounder but part of a true clinical picture, because SE may be caused by underlying diseases. Thus, high CCI may reflect underlying morbidity that started years earlier, with SE appearing only at an advanced stage.

Our group previously published a retrospective chart review of hospitalized patients with SE.^11^ The current study differs substantially from the previous one in several key aspects. First, it uses a nationwide, population-based matched cohort design (SE cases vs non-SE controls), which enhances subject heterogeneity, geographic diversity, and generalizability compared with the previous observational study limited to 3 hospitals in the Jerusalem area. Second, it includes both inpatients and outpatients, whereas the previous study was restricted to hospitalized patients. Third, it evaluates the long-term (5-year) prognostic implications of SE in the general population (eg, risks of malignancy, autoimmune disease, thromboembolism, and mortality via Cox models), rather than describing in-hospital etiologies and cross-sectional characteristics.

Our study found that SE was associated with increased risk of allergic disorders in children but not in adults. One plausible explanation to this discrepancy is the predominance of T_H_2-skewed immunity in early life, which gradually shifts with age. In a study of 256 nonatopic healthy individuals and 87 allergic individuals, the T_H_1/T_H_2 balance was shown to change progressively during growth, with higher T_H_1/T_H_2 ratios observed in people in their twenties and thirties compared with younger age groups.^17^ Early and repeated exposure to food allergens during infancy and childhood may further enhance type 2 responses and eosinophil activation, which could help explain why SE was associated with allergic diseases in children but not in adults in our study.

The most notable finding in our study is the characterization of cancer risk in both adults and children with SE. Although the overall 5-year risk of developing any type of cancer was elevated in both cohorts, distinct patterns emerged: children with SE were more likely to develop solid tumors, whereas adults exhibited a higher risk of hematologic malignancies. When leukemia subtypes were examined separately, myelogenous leukemias occurred more frequently among individuals with SE, whereas lymphocytic leukemias were rare in both cohorts. Although these descriptive patterns may suggest stronger alignment of SE with myeloid proliferative disorders, the number of events was small, and estimates should be interpreted cautiously.

Paraneoplastic SE was previously reported.^14^^,^^15^^,^^18^^,^^19^ However, observational studies evaluating the broader association between SE and cancer are scarce. In one study, among 239 hospitalized patients with SE, 42.25% were diagnosed with cancer, predominantly hematologic malignancies.^11^ Our study demonstrates that SE may serve not only as a marker of coexisting cancer but also as a predictive biomarker for future cancer diagnosis, in both children and adults.

Several mechanisms have been proposed linking eosinophilia to tumorigenesis. These include induction of angiogenesis and metastases, TME alterations, and direct tissue infiltration that leads to chronic inflammation, fibrosis, and remodeling. Eosinophils were found to promote angiogenesis and metastases via tumor-associated tissue eosinophilia (TATE) in head and neck squamous cell carcinoma. These tumors exhibited aggressive features: tumor growth, nodal spread, and tissue invasion.^20^

In terms of TME modulation, eosinophils appear to play a dual role, both promoting and inhibiting tumor development. On one hand, activated eosinophils can release cytotoxic granules capable of killing tumor cells. On the other hand, immune dysregulation and T_H_1/T_H_2 repolarization^8^^,^^21^ supports tumor growth.^22^ This dual role of eosinophilia and TATE is also supported by previous studies, which found that tumors lacking TATE and eosinophilia exhibited more aggressive features in colorectal^23^ and breast cancer.^24^

Our findings also indicate an elevated risk of TED in adults with SE. Several case reports have described thrombotic complications in the context of eosinophilia.^25^, ^26^, ^27^ In addition, a study demonstrated that persistent eosinophilia was linked to a shorter time to recurrence of venous thrombosis, suggesting that resolution of eosinophilia may reduce the risk of future TED.^28^ This may be attributed to the prothrombotic properties of eosinophil granule proteins, including major basic protein, eosinophil peroxidase, and eosinophil cationic protein, which can injure vascular endothelium. Other proposed mechanisms involve chronic eosinophilic vasculitis and platelet activation through eosinophil-induced upregulation of CD40 ligand.^29^ Moreover, eosinophil-secreted cationic proteins may have an inhibitory effect on antithrombotic proteins, such as thrombomodulin.^30^

We also found that children and adults with SE had an increased risk of developing AID. Eosinophilia can be found in several AIDs, including inflammatory bowel disease and eosinophilic granulomatosis with polyangiitis.^31^ Notably, although SE is also a recognized feature of certain primary immune regulatory disorders,^32^ our multivariable analysis adjusted for and excluded inborn errors of immunity, to minimize confounding. The observed association between SE and AID may be explained by eosinophil-driven mechanisms, such as chronic tissue inflammation leading to fibrosis and remodeling, and antibody-dependent cellular cytotoxicity that contributes to self-tissue injury.^31^

Finally, our findings reveal a concerning 8-fold increase in mortality in children and a 2-fold increase in adults with SE. This is supported by a study showing that SE was missed or misdiagnosed as an infection in 28% of hospitalized cases.^11^ Previous reports on mortality in individuals with SE have primarily focused on adult populations.^33^^,^^34^ Our study describes a substantial increase in mortality risk among children with SE. This observation contrasts with an earlier single-center report suggesting low mortality rates in children with hypereosinophilia. However, these previous findings may have been influenced by referral bias, because subjects were treated at a specialized tertiary care center.^35^

**Fig 4:**
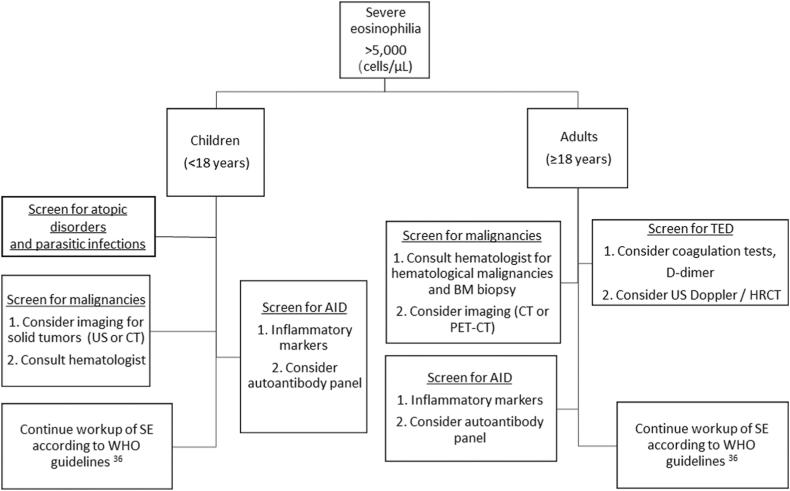
Suggested diagnostic workup of patients with SE. *CT*, Computed tomography; *HRCT*, high-resolution computed tomography; *PET*, positron emission tomography; *US*, ultrasound; *WHO*, World Health Organization.

The implications for clinical practice indicate that SE cannot be managed with a uniform, “one-size-fits-all” protocol. Diagnostic pathways must be stratified by age. In adults, evaluation should prioritize exclusion of hematologic malignancies, autoimmune diseases, and coagulopathic risks. In children, the focus should instead be on ruling out solid tumors and severe atopic or autoimmune disorders. Fig 4 summarizes our additional recommendations for the diagnostic approach to SE, complementing the current World Health Organization guidelines.^36^

In children, the presence of SE necessitates a diagnostic workup that differs markedly from standard hematologic protocols. Although SE is often clinically associated with blood cancers, our data show that pediatric SE is a specific warning sign for solid tumors, particularly skin malignancies. Therefore, diagnostic evaluation in children should prioritize imaging and screening for solid neoplasms, rather than focusing solely on bone marrow pathology. The differential diagnosis should also include a broad assessment for autoimmune and allergic disorders, which are highly prevalent in this population. Given the 8-fold increase in mortality observed, once parasitic infections and atopic disorders are excluded, SE in a child should be treated as an ominous prognostic marker, warranting aggressive diagnostic evaluation even in the absence of other systemic symptoms.

In adults with SE, the primary concern remains hematologic malignancies such as leukemia, lymphoma, and multiple myeloma. However, the workup should extend beyond malignancy to include targeted assessment for TME and AID, both of which are strongly associated with SE in adults. In this population, SE appears to function as a marker of prothrombotic and inflammatory risk. Clinicians should therefore incorporate prophylactic vascular assessment and autoimmune screening into the standard diagnostic evaluation, rather than limiting the workup to oncologic causes.

The main strength of this study is its real-world, population-based design, though it has several limitations. Despite careful adjustment for demographic variables, residual unmeasured confounders, such as lifestyle, genetic predisposition, and medication use, remain possible. Furthermore, the ethnicity variable available in our study data sets provides only broad groupings (Jews, Arabs, Others) and does not capture substantial within-group heterogeneity. Although this classification is standard in Israeli epidemiological research and was used solely for confounding control, residual confounding related to unmeasured sociodemographic or cultural differences may persist. More granular ethnicity information was not uniformly available and therefore could not be incorporated. In addition, parasitic infections, a known cause of SE, were not systematically measured in this study. In clinical practice, parasitic infections are often underreported, because many patients receive empirical treatment without confirmatory laboratory testing due to the high cost and limited availability of specific diagnostic assays. Nevertheless, it is important to note that the prevalence of severe parasitic infections in Israel is considered low.^37^ In particular, penetrating SE-causing parasites, such as *Strongyloides*, have rarely been reported in epidemiologic studies on parasitic infections in Israel.^38^ Another potential limitation is misclassification bias. Our definition of SE was based on a single documented blood test, which may have included both transient and persistent eosinophilia. To acknowledge this, we consistently referred to the exposure as “SE” rather than “persistent eosinophilia,” recognizing that some cases may have resolved over time. Furthermore, the possibility of ascertainment bias must be considered. Individuals with SE may undergo more frequent diagnostic evaluations, potentially leading to earlier or more frequent detection of adverse outcomes compared with non-SE subjects. Importantly, reverse causation should also be considered; that is, SE may have been a consequence rather than a cause of autoimmune disease or cancer. To address this, we excluded all patients with preexisting outcomes before the index date and applied a 6-month lag period following the diagnosis of SE. Nevertheless, some degree of reverse causation may still persist. Another limitation is that analyses of leukemia subtypes were constrained by small numbers of events, which reduced the precision of subtype-specific risk estimates and precluded fully adjusted multivariable models. Consequently, these findings should be interpreted as exploratory. Lastly, the generalizability of our findings may be questioned, because the study was conducted within a single national health care system. However, CHS is Israel’s largest health care provider with nearly half of its population insured. Therefore, the study population includes diverse ethnic groups, including Ashkenazi and Sephardic Jews, Arabs, and other minorities, all with equal access to CHS under national health care law.

### Conclusion

Our findings suggest that SE should prompt clinicians to consider a diagnostic approach, even in the absence of symptoms. Although parasitic and allergic causes remain important, the potential for underlying malignancies, AID, and TED warrants further attention. Future studies should validate our findings, evaluate the effectiveness of our SE management recommendations, and assess the cost-benefit ratio of targeted diagnostic workup strategies.

## Disclosure statement

This study was supported (in part) by a grant from the Israeli Association of Allergy and Clinical Immunology.

Disclosure of potential conflict of interest: The authors declare that they have no relevant conflicts of interest.

Ethical review of the study: The study protocol was approved by CHS Institutional Review Board (IRB # KMC-0106-23). Confidentiality complied with ethical guidelines for human subject research.

Data sharing statement: Data are available on request from the corresponding author.
