# Supplementary material for: Severe eosinophilia and risk of major disease and mortality: A nationwide cohort study of children and adults
Source: J Allergy Clin Immunol Glob. 2026 Feb 4;5(3):100654. doi: 10.1016/j.jacig.2026.100654 (PMC12968414; doi:10.1016/j.jacig.2026.100654)
Supplement: Supplementary Tables [file mmc1.docx]

| **Cohort** | **Outcome** | **Study group** | | | | | | |
| --- | --- | --- | --- | --- | --- | --- | --- | --- |
|  |  | **Total** | | **Non-SE subjects** | | **Severe eosinophilia** | | **p-value** |
|  |  | **N** | **%** | **N** | **%** | **N** | **%** |  |
| **Adults**  **(≥18 years)** | **Cancer (Any)** | 3738 | 12.76 | 2982 | 11.13 | 756 | 30.28 | <0.001* |
|  | **Solid Cancer** | 2925 | 10.27 | 2594 | 9.82 | 331 | 15.97 | <0.001* |
|  | **Hematologic Cancer** | 813 | 3.08 | 388 | 1.60 | 425 | 19.62 | <0.001* |
|  | **Autoimmune disease** | 4633 | 15.82 | 3960 | 14.78 | 673 | 26.95 | <0.001* |
|  | **Autoinflammatory disease** | 116 | 0.40 | 99 | 0.37 | 17 | 0.68 | .018* |
|  | **Immunodeficiency** | 90 | 0.31 | 66 | 0.25 | 24 | 0.96 | <0.001* |
|  | **Any Allergy** | 10339 | 35.30 | 9060 | 33.82 | 1279 | 51.22 | <0.001* |
|  | **Thromboembolic disease** | 689 | 2.35 | 566 | 2.11 | 123 | 4.93 | <0.001* |
|  | **Septic shock** | 48 | 0.16 | 14 | 0.05 | 34 | 1.36 | <0.001* |
|  | **Prior splenectomy** | 75 | 0.26 | 30 | 0.11 | 45 | 1.80 | <0.001* |
|  | **Adrenal sufficiency** | 25 | 0.09 | 20 | 0.07 | 5 | 0.20 | .040* |
| **Children**  **(<18 years)** | **Cancer (Any)** | 101 | 0.75 | 16 | 0.13 | 85 | 6.42 | <0.001* |
|  | **Solid Cancer** | 32 | 0.24 | 7 | 0.06 | 25 | 1.98 | <0.001* |
|  | **Hematologic Cancer** | 69 | 0.51 | 9 | 0.07 | 60 | 4.62 | <0.001* |
|  | **Autoimmune disease** | 847 | 6.26 | 657 | 5.38 | 190 | 14.34 | <0.001* |
|  | **Autoinflammatory disease** | 31 | 0.23 | 27 | 0.22 | 4 | 0.30 | 0.559 |
|  | **Immunodeficiency** | 69 | 0.51 | 29 | 0.24 | 40 | 3.02 | <0.001* |
|  | **Any Allergy** | 3044 | 22.48 | 2635 | 21.58 | 409 | 30.87 | <0.001* |
|  | **Thromboembolic disease** | 8 | 0.06 | 0 | 0.00 | 8 | 0.60 | <0.001* |
|  | **Septic shock** | 9 | 0.07 | 0 | 0.00 | 9 | 0.68 | <0.001* |
|  | **Prior splenectomy** | 13 | 0.10 | 0 | 0.00 | 13 | 0.98 | <0.001* |

**Repository Table 1: Pre-index frequencies of outcomes in subjects with severe eosinophilia vs. non-SE subjects**

SE- Severe eosinophilia; * The Chi-square statistic is significant at the 0.05 level.

| **Cancer group** | | **Study group** | | | | | |
| --- | --- | --- | --- | --- | --- | --- | --- |
|  |  | **Total** | | **Non-SE subjects** | | **Severe eosinophilia** | |
|  |  | N | % | N | % | N | % |
| Cancer group (pre-index*) | Lymphoma | 329 | 40.47 | 249 | 64.18 | 80 | 18.82 |
|  | Leukemia | 399 | 49.08 | 78 | 20.10 | 321 | 75.53 |
|  | Multiple Myeloma | 85 | 10.46 | 61 | 15.72 | 24 | 5.65 |
| Cancer group (post-index*) | Lymphoma | 87 | 50.58 | 76 | 53.15 | 11 | 37.93 |
|  | Leukemia | 51 | 29.65 | 37 | 25.87 | 14 | 48.28 |
|  | Multiple Myeloma | 34 | 19.77 | 30 | 20.98 | 4 | 13.79 |

**Repository table 2: Frequencies of hematologic cancer types in the adult cohort**

SE- Severe eosinophilia; N- Number of patients. * Eosinophil testing date was designated as the index date.

| **Cancer group** | | **Study group** | | | | | |
| --- | --- | --- | --- | --- | --- | --- | --- |
|  |  | **Total** | | **Non-SE subjects** | | **Severe eosinophilia** | |
|  |  | N | % | N | % | N | % |
| Cancer group (pre-index*) | Digestive system | 104 | 3.56 | 94 | 3.62 | 10 | 3.02 |
|  | Brain | 5 | 0.17 | 3 | 0.12 | 2 | 0.60 |
|  | Skin | 2087 | 71.35 | 1900 | 73.25 | 187 | 56.50 |
|  | Liver | 41 | 1.40 | 26 | 1.00 | 15 | 4.53 |
|  | Breast | 37 | 1.26 | 34 | 1.31 | 3 | 0.91 |
|  | Other Solid | 651 | 22.26 | 537 | 20.70 | 114 | 34.44 |
| Cancer group (post-index*) | Digestive system | 50 | 3.81 | 46 | 3.77 | 4 | 4.35 |
|  | Brain | 17 | 1.30 | 16 | 1.31 | 1 | 1.09 |
|  | Skin | 845 | 64.45 | 787 | 64.56 | 58 | 63.04 |
|  | Lung | 6 | 0.46 | 6 | 0.49 | 0 | 0.00 |
|  | Liver | 69 | 5.26 | 65 | 5.33 | 4 | 4.35 |
|  | Breast | 17 | 1.30 | 17 | 1.39 | 0 | 0.00 |
|  | Other Solid | 307 | 23.42 | 282 | 23.13 | 25 | 27.17 |

**Repository Table 3: Distribution of Solid Tumors Among Adults**

SE- Severe eosinophilia; N- Number of patients; * Eosinophil testing date was designated as the index date.

| **Cancer group** | | **Study group** | | | | | |
| --- | --- | --- | --- | --- | --- | --- | --- |
|  |  | **Total** | | **Non-SE subjects** | | **Severe eosinophilia** | |
|  |  | N | % | N | % | N | % |
| Cancer group (pre-index*) | Lymphoma | 10 | 14.49 | 4 | 44.44 | 6 | 10.00 |
|  | Leukemia | 59 | 85.51 | 5 | 55.56 | 54 | 90.00 |
| Cancer group (post-index*) | Lymphoma | 9 | 81.82 | 7 | 87.50 | 2 | 66.67 |
|  | Leukemia | 2 | 18.18 | 1 | 12.50 | 1 | 33.33 |

**Repository table 4: Frequencies of hematologic malignancies in the pediatric cohort**

SE- Severe eosinophilia; N- Number of patients. * Eosinophil testing date was designated as the index date.

| **Cancer group** | | **Study group** | | | | | |
| --- | --- | --- | --- | --- | --- | --- | --- |
|  |  | **Total** | | **Non-SE subjects** | | **Severe eosinophilia** | |
|  |  | **N** | **%** | **N** | **%** | **N** | **%** |
| Cancer group (pre-index*) | Skin | 9 | 28.13 | 4 | 57.14 | 5 | 20.00 |
|  | Liver | 1 | 3.13 | 0 | 0.00 | 1 | 4.00 |
|  | Other Solid | 22 | 68.75 | 3 | 42.86 | 19 | 76.00 |
| Cancer group (post-index*) | Skin | 2 | 25.00 | 2 | 100.00 | 0 | 0.00 |
|  | Other Solid | 6 | 75.00 | 0 | 0.00 | 6 | 100.00 |

**Repository table 5: Distribution of solid tumors among children**

SE- Severe eosinophilia; N- Number of patients. * Eosinophil testing date was designated as the index date.

| **Cohort** | **Leukemia subtype** | **Total** | | **Non-SE subjects** | | **Severe eosinophilia** | |
| --- | --- | --- | --- | --- | --- | --- | --- |
|  |  | N | % | N | % | N | % |
| Adults  (≥18 years) | No Leukemia | 29238 | 99.83 | 26755 | 99.86 | 2483 | 99.44 |
|  | Lymphoid Leukemia | 11 | 0.04 | 8 | 0.03 | 3 | 0.12 |
|  | Myeloid Leukemia | 9 | 0.03 | 6 | 0.02 | 3 | 0.12 |
|  | Other/Unspecified Leukemia | 31 | 0.11 | 23 | 0.09 | 8 | 0.32 |
| Children  (<18 years) | No Leukemia | 13536 | 99.99 | 12212 | 99.99% | 1324 | 99.92% |
|  | Lymphoid Leukemia | 1 | 0.01 | 1 | 0.01 | 0 | 0.00 |
|  | Other/Unspecified Leukemia | 1 | 0.01 | 0 | 0.00 | 1 | 0.08 |

**Repository Table 6: Frequencies of leukemia subtypes as outcome in children and adults with severe eosinophilia.**

SE- Severe eosinophilia.

| **Outcome/ Variable** | **HR (95% CI)** | P value |
| --- | --- | --- |
| Study group | 2.163 (1.257-3.721) | 0.005* |
| Elevated CRP (>0.5 mg/dL) | 1.905 (1.119-3.243) | 0.018* |
| Age at Index date, years | 1.037 (1.014- 1.062) | 0.002* |
| Sex | 0.813 (0.508-1.302) | 0.389 |
| SES (3L) | - | 0.116 |
| SES (3L) (Medium) | 0.985 (0.565-1.717) | 0.956 |
| SES (3L) (High) | 0.536 (0.269-1.069) | 0.077 |
| Ethnicity | - | 0.355 |
| Ethnicity (Arabic) | 1.585 (0.764-3.288) | 0.216 |
| Ethnicity (Other) | 0.669 (0.161-2.789) | 0.581 |
| Born in Israel | 0.897 (0.497-1.622) | 0.720 |
| BMI | 1.030 (0.992-1.070) | 0.127 |
| CCI | 1.057 (0.940-1.189) | 0.356 |
| AID (Pre-Index) | 1.127 (0.685-1.854) | 0.637 |
| Autoinflammatory disease (Pre-Index) | 0.000 (0.000---) | 0.960 |
| Immunodeficiency (Pre-Index) | 2.632 (0.358-19.324) | 0.341 |
| Any Allergy (Pre-Index) | 0.794 (0.495-1.275) | 0.340 |
| Thromboembolic disease (Pre-Index) | 1.464 (0.625-3.427) | 0.380 |

**Repository table 7: Risk of developing hematologic cancer in adult subjects with elevated CRP and severe eosinophilia**

CRP- C-reactive protein; SES- Socio-Economic Score; BMI- Body mass index; CCI- Charlson Comorbidity Index; AID- Autoimmune disorders; HR- Hazard ratios; CI- Confidence interval. * The Chi-square statistic is significant at the 0.05 level

| **P value** | **Facility where eosinophil testing was performed** | | | | | | **Outcome** | **Study's cohort** |
| --- | --- | --- | --- | --- | --- | --- | --- | --- |
|  | **Hospitalized** | | | **Outpatients** | | |  |  |
|  | **% Within outcome (post-index)** | **% Within performing facility** | **N** | **% Within outcome (post-index)** | **% Within performing facility** | **N** |  |  |
| 0.615 | 62.2 | 17.8 | 138 | 37.8 | 19.0 | 84 | **Allergy (any)** | **Adults (≥18 years)** |
| 0.671 | 58.7 | 8.3 | 91 | 41.3 | 8.8 | 64 | **AID** |  |
| 0.409 | 55.4 | 6.5 | 67 | 44.6 | 7.6 | 54 | **Cancer (any)** |  |
| 0.051 | 48.9 | 4.5 | 45 | 51.1 | 6.6 | 47 | **Solid cancer** |  |
| 0.070 | 75.9 | 2.2 | 22 | 24.1 | 1.0 | 7 | **Hematological cancer** |  |
| 0.665 | 63.5 | 2.8 | 40 | 36.5 | 2.5 | 23 | **Thromboembolic disorders** |  |
| 0.500 | 69.4 | 24.4 | 159 | 30.6 | 26.5 | 70 | **Allergy (any)** | **Children (<18 years)** |
| 0.044* | 76.8 | 9.5 | 73 | 23.2 | 6.0 | 22 | **AID** |  |
| 0.036* | 100 | 1.1 | 9 | 0.0 | 0.0 | 0 | **Cancer (any)** |  |
| 0.087 | 100 | 0.7 | 6 | 0.0 | 0.0 | 0 | **Solid cancer** |  |
| 0.226 | 100 | 0.4 | 3 | 0.0 | 0.0 | 0 | **Hematological cancer** |  |

**Repository Table 8: Post-index outcome frequencies by eosinophil testing site (Hospitalized vs. Outpatients)**

AID- Autoimmune disorders; * The Chi-square statistic is significant at the 0.05 level.
